# Molecular docking analysis of *Salvia sclarea* flower extracts evaluated for protein target affinity based on different extraction methods

**DOI:** 10.1002/fsn3.4467

**Published:** 2024-09-15

**Authors:** Emine Incilay Torunoglu, Erdi Can Aytar, Alper Durmaz

**Affiliations:** ^1^ Department of Medical Biochemistry, Faculty of Medicine Necmettin Erbakan University Konya Turkey; ^2^ Department of Horticulture, Faculty of Agriculture Usak University Uşak Turkey; ^3^ Ali Nihat Gokyigit Botanical Garden Application and Research Center Artvin Coruh University Artvin Turkey

**Keywords:** antioxidant activity, GC–MS analysis, maceration, molecular docking, *Salvia sclarea*

## Abstract

This study evaluates the antioxidant activity and total phenolic, flavonoid, flavonol, and tannin contents of extracts obtained from the flower parts of *Salvia sclarea* using different extraction methods, such as maceration and rotavapor (RE). The results indicate that the maceration method yields extracts with higher antioxidant activity compared to the rotavapor method (DPPH IC_50_: 260.70 ± 65.41 mg/mL vs. 345.48 ± 27.91 mg/mL). Additionally, the maceration method produces higher values in terms of total phenolic (26.4 ± 7.78 mg GAE/g extract), flavonoid (10.44 ± 0.21 mg QE/g extract), flavonol (9.20 ± 0.84 mg QE/g extract), and tannin contents (8.36 ± 0.39 mg GAE/g extract), whereas the RE method shows lower values (phenolics: 19.8 ± 3.31 mg GAE/g extract, flavonoids: 10.35 ± 0.35 mg QE/g extract, flavanol's: 5.45 ± 0.01 mg QE/g extract, and tannins: 7.72 ± 0.10 mg GAE/g extract). The gas chromatography–mass spectrometry (GC–MS) analysis of the extracts identified various bioactive compounds. Molecular docking studies revealed that Terpinen‐4‐ol obtained through maceration exhibits a strong binding affinity to a specific protein target, indicating potential biological activity. The predicted pharmacokinetic (ADME (absorption, distribution, metabolism, and excretion)) parameters for Terpinen‐4‐ol suggest favorable absorption and central nervous system (CNS) penetration, while Squalene obtained through maceration shows different pharmacokinetic properties. *S. sclarea* highlights significant antioxidant potential and provides insight into the bioactive compounds obtained through different extraction methods. To further investigate the potential biological interactions arising from these differences, molecular docking studies were conducted.

## Graphical abstract

This study demonstrated that extracts from *Salvia sclarea* flowers obtained through maceration exhibited higher antioxidant activity and greater contents of phenolics, flavonoids, flavonols, and tannins compared to those obtained via rotavapor (RE). Gas chromatography–mass spectrometry (GC–MS) analysis identified various bioactive compounds, with molecular docking revealing Terpinen‐4‐ol's strong binding affinity and favorable pharmacokinetic properties. These findings suggest that maceration may enhance the bioactivity of *S. sclarea* extracts.

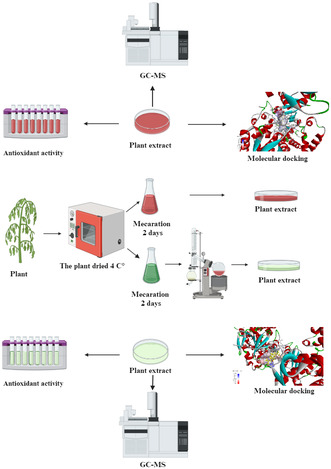

## 1 INTRODUCTION

**FIGURE 1:**
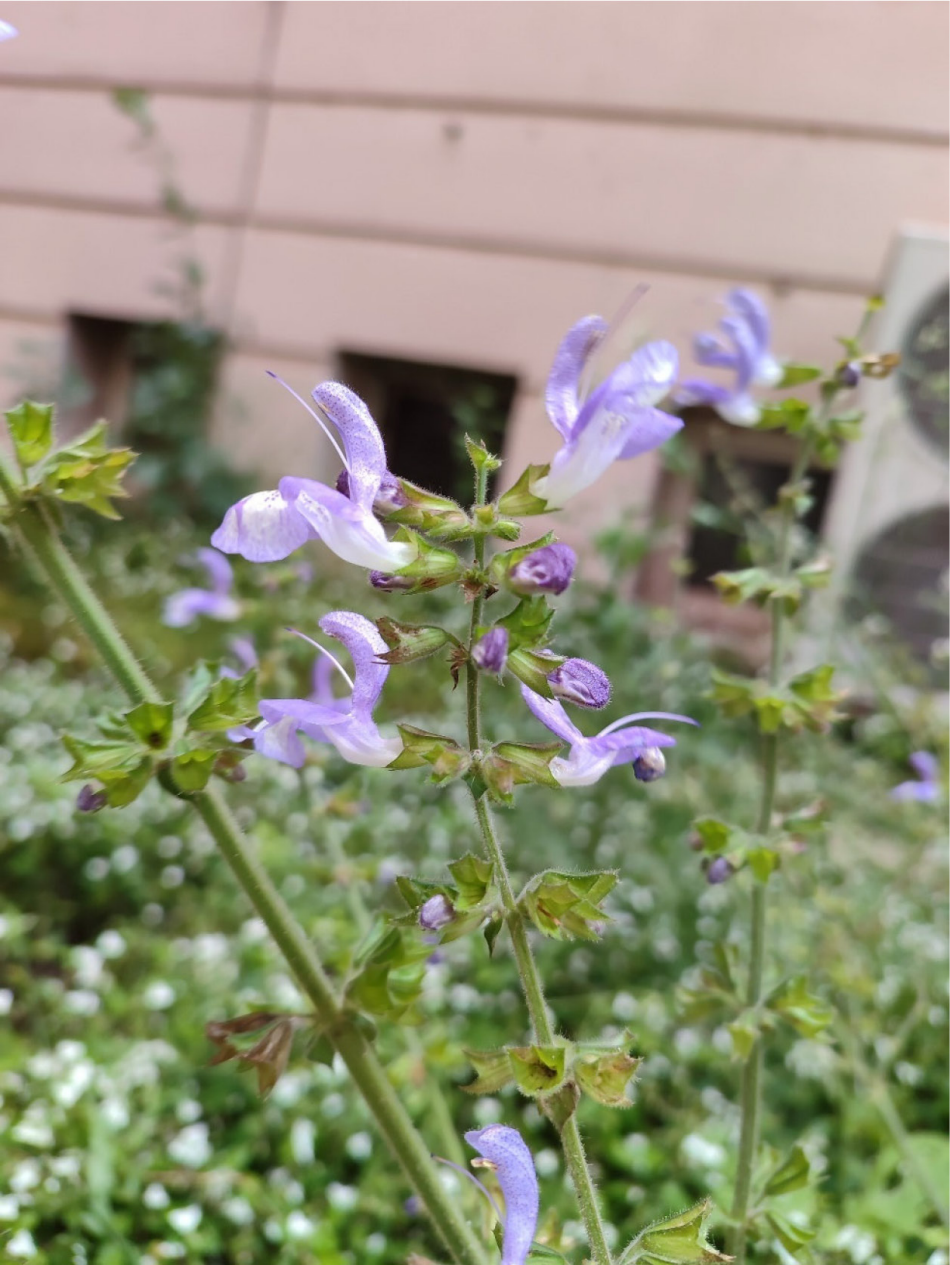
*Salvia sclarea* plant morphological structure.

*Salvia sclarea*, belonging to the Lamiaceae family, is commonly known as Clary sage. It is a shrub that reaches heights of 100–120 cm and is naturally found in Europe, Asia, and the Caucasus regions (Figure 1). This plant tends to grow in stony and clayey slopes (Kačániová et al., 2023; Zhussupova et al., 2022). *S. sclarea* is also recognized by other names such as “clear eye” and “hairy sage,” in addition to “Clary sage” (Onder et al., 2022). Moreover, *S. sclarea* finds frequent use in the cosmetic industry (Ben Taârit et al., 2012). *S. sclarea* seeds are known to be rich in fatty acids and serve as a good source of antioxidants (Asadi et al., 2010; Tepe et al., 2006). Additionally, *S. sclarea* has been reported to have neuroprotective (Kuźma et al., 2007), antimicrobial (Seol et al., 2010), antidepressant (Tuttolomondo et al., 2020), and anticancer (Noori et al., 2010) effects. These academic studies emphasize the strong antioxidant properties of *S. sclarea* and its potential role as a natural antioxidant source. The *Salvia* genus is one of the most important members of the Lamiaceae family, with over 1000 species, commonly found in various regions, particularly within the Mediterranean and Asian regions (Henríquez et al., 2023). Turkey hosts a rich diversity of the *Salvia* genus, boasting 100 species, 57 of which are exclusive to the region. *Salvia* species in Turkey are recognized for their biological activities, including unique antiviral, antibacterial, antifungal, and antioxidant properties (Maral, 2023). The studies have reported that *S. sclarea* exhibits antifungal activity (Yüce et al., 2014).

Antioxidants are defined as chemical mechanisms that protect cells from the harmful effects of free radicals. Antioxidants are compounds that protect living organisms against harm arising from lipid peroxidation, protein damage, and DNA strand breakage caused by the production of reactive oxygen species (ROS). Among the compounds with protective effects, phenolic acids, polyphenolic substances, and flavonoids, among various plant‐derived compounds, are known to exhibit these protective antioxidant properties (Kalpoutzakis et al., 2023). Phenolic compounds derived from plants also possess antioxidant properties. These antioxidant compounds exhibit various activities, including anti‐atherosclerotic, anti‐inflammatory, antitumor, anticarcinogenic, antimutagenic, antiviral, antibacterial, and anti‐neurodegenerative effects (Güzel, 2023). These antioxidants help prevent cell damage by inhibiting or preventing the harmful effects of free radicals, thus aiding in the prevention of cell damage (Ariani et al., 2023). These antioxidant compounds are typically produced as secondary metabolites in plants and are referred to as phytochemicals. Phenolic compounds, known as one of the fundamental secondary metabolites in plants, can exhibit effective antioxidant functions when found in high amounts in plants (Nwozo et al., 2023; Zengin et al., 2024).

The superfamily of Cytochrome P450 (CYP) currently comprises approximately 9000 proteins forming over 800 families. These enzymes catalyze the monooxygenation of a wide range of compounds and primarily serve two functions. They provide biodefense by detoxifying xenobiotics and producing antibiotics, as well as participate in the biosynthesis of crucial endogenous molecules, particularly steroids. Based on these dual functions, sterol 14α‐demethylases (CYP51) belong to the second group of P450s. The specific inhibition of protozoal CYP51 may play a role in the treatment of human trypanosomiasis (Lepesheva et al., 2008).

The primary objective of this study is to compare the 2,2‐diphenyl‐1‐picrylhydrazyl (DPPH), total phenol, flavonoid, flavonol, and tannin contents of extracts obtained from the flower part of *S. sclarea* using maceration extraction (ME) and rotavapor extraction (RE) methods. Additionally, this study aims to elucidate the differences in secondary metabolites resulting from these distinct extraction methods through GC–MS analysis. To further investigate the potential biological interactions arising from these differences, a molecular docking study was conducted.

## 2 MATERIALS AND METHODS

### 2.1 Plant extraction

#### 2.1.1 Maceration extraction (ME)

*Salvia sclarea* plants were collected from the flower parts in July on the campus of Ondokuz Mayıs University, Samsun, Turkiye. The flowers of *S. sclarea* were initially dried in an oven at 40°C and then ground into a powder using a blender. The maceration method recommended by Aytar (2024) was applied for extraction, where 0.04 g of the powder was extracted with 40 mL of 80% methanol at 35°C for a duration of 24 h.

#### 2.1.2 Rotavapor extraction (RE)

Flowers dried at 40°C (20 g) were extracted with (Aytar, 2024) methanol for 2 days at room temperature in a dark environment. The obtained methanolic extracts were filtered through filter paper. After filtration, the solvent was evaporated in a rotary evaporator (Heidolph, Germany) at 40°C under reduced pressure, and the solid extracts were stored at +4°C until further use.

### 2.2 Analysis of secondary metabolites using spectroscopy

#### 2.2.1 Total phenolic content

The determination of the total phenolic content in the extract involved the application of the Folin–Ciocalteu method with some adjustments, following the protocol presented in (Singleton & Rossi, 1965). Within the scope of this research, 200 μL of the extract (1 mg/mL) was blended with 200 μL of Folin–Ciocalteu reagent, which had been appropriately diluted (1:1 with distilled water). After allowing the reaction mixture to incubate for 3 min at room temperature, an addition of 1 mL of a 2% sodium carbonate (Na_2_CO_3_) solution followed. Following an hour of incubation under dark conditions at room temperature, measurement of the absorbance took place at 760 nm using an ultraviolet (UV) spectrophotometer (Thermo Scientific Varioskan). The quantification of total phenolic content was articulated in terms of gallic acid equivalent (GAE), represented as milligrams per gram of dried extract (mg GAE/g extract). All procedures were carried out in triplicate.

#### 2.2.2 Total flavonoid content

The determination of the total flavonoid content in the extracts was carried out using the aluminum chloride (AlCl_3_) method with some adjustments, following the procedure outlined in (Osuna‐Ruiz et al., 2016). Within the context of this study, 6.4 mL of distilled water was combined with 1 mL of the extracts. Subsequently, 0.3 mL of sodium nitrite (NaNO_2_) (5%) was introduced into the mixture and allowed to stand for 5 min. Following this, 0.3 mL of AlCl_3_ (10%) was added, and the solution was subjected to a six‐minute incubation period. Afterward, 2 mL of sodium hydroxide (NaOH) (1 M) was incorporated into the solution, which was then left at room temperature for 30 min. An ultraviolet (UV) spectrophotometer was used to measure the absorbance at 510 nm. The quantification of total flavonoid content was expressed as quercetin equivalent (QE), represented as milligrams per gram of dried extract (mg QE/g). All procedures were replicated three times.

#### 2.2.3 Total flavanol content

The aluminum chloride method (Mahmoudi et al., 2023) was employed to determine the total flavanol content. In a nutshell, 1 mL of the extracts (1 mg/mL) was combined with 2 mL of AlCl_3_ solution (2%). Following this, 3 mL of sodium acetate solution (5%) was introduced. The mixture was subsequently left at room temperature in a dark environment for 30 min. After the incubation period, the sample's absorbance was measured at 415 nm, using quercetin as the reference standard. The quantification of total flavonol content was conveyed as quercetin equivalents (mg QE/g).

#### 2.2.4 Total tannin content

The determination of the total tannin content followed the procedure outlined in (Lou et al., 2014) using the Folin–Ciocalteu reagent. A calibration curve was constructed using various concentrations of gallic acid (GA) dissolved in methanol. For the samples, 100 μL of a 5‐fold diluted sample in methanol was introduced into a test tube. Similarly, for both the standards and samples, 500 μL of a 10‐fold diluted Folin–Ciocalteu reagent in water and 2 mL of aqueous sodium carbonate solution (4%, w/v) were added. The resulting mixture was agitated and then allowed to incubate for 30 min in darkness at room temperature. Following incubation, the absorbance of both standards and samples was recorded at 760 nm. The quantification of total tannin content was expressed as gallic acid equivalent (GAE), represented in milligrams per gram of dried extract (mg GAE/g extract). All procedures were conducted in triplicate.

### 2.3 DPPH radical scavenging assay

The DPPH radical scavenging assay was employed to evaluate the extracts' ability to scavenge free radicals, and a comparison was made with the half maximal inhibitory concentration (IC_50_) value of the synthetic antioxidant ascorbic acid. This assay was conducted with slight modifications based on the approach outlined in (Braca et al., 2001). In this assay, 1 mL of the extract at various concentrations was mixed with a methanol solution containing the DPPH radical (0.1 mM) within a tube. After a 30‐min incubation period at room temperature in the absence of light, the absorbance was gauged at 517 nm using an ultraviolet (UV) spectrometer (Thermo Scientific Varioskan Flash) against a blank. Ascorbic acid was utilized as a reference standard. The percentage of DPPH radical scavenging effect by the extract was calculated using the equation: DPPH scavenging activity (% inhibition) = [(A_control_—A_sample_)/A_control_] × 100, where A_control_ represents the absorbance of the control and A_sample_ signifies the absorbance of the reaction mixture containing the extract. To determine the concentration of the extract required to elicit a 50% reduction in the initial DPPH concentration, a concentration curve plotting the extract concentration against the percentage of inhibition was generated, and the IC_50_ value was derived through linear regression analysis. A lower IC_50_ value indicates stronger antioxidant activity. All measurements were conducted in triplicate.

### 2.4 GC–MS analysis

*Salvia sclarea* flowers were dried at 40°C, pulverized, and subjected to extraction using the ME method recommended by (Aytar, 2024). The extraction involved dissolving 0.04 g of the powder in 40 mL of 80% methanol at 35°C for 24 h. Additionally, flowers (20 g) dried at 40°C were extracted with methanol (1:4 w/v) using the ME method over complementary web tool SwissADME3 days at room temperature in the dark. RE was filtered, evaporated, and stored at +4°C. Optimization structures were analyzed through GC–MS using SHIMADZU GCMS‐QP2010 Mass Spectrometry with an Rxi‐5MS column. Samples were centrifuged, and the supernatant was used for GC–MS analysis. The analysis included an electron ionization (EI) system, helium gas with a constant flow rate, and specific temperature settings. The National Institute of Standards and Technology (NIST) Standard Reference database was employed for analysis.

### 2.5 Molecular docking studies

Molecules were drawn in the ChemDraw Ultra 18.0 program, and their minimal energy forms were obtained in the Chem3D 18.0 program and saved in Mol2 format. The Protein Data Bank (PDB) was used to record the enzymes. The 14α‐demethylase (CYP51) structure PDB‐ID 3LD6 (2.80 Å) was chosen and preserved in PDB format. Molecule–enzyme interactions using AutoDock Vina 1.5.7 software and binding energies (kcal/mol) were determined (Trott & Olson, 2010). Two‐dimensional (2D) and three‐dimensional (3D) visuals are demonstrated by BIOVIA Discovery Studio Visualizer software (Biovia, 2019).

### 2.6 In silico and ADME and drug‐likeness prediction

The in silico ADME screening and drug‐likeness evaluation were conducted utilizing the complementary web tool SwissADME, developed by the SIB Swiss Institute of Bioinformatics and accessible at www.swissadme.ch (Daina et al., 2017). Compounds with notable binding energy scores underwent this segment of the screening process. Basic physicochemical properties, such as molecular weight (MW), molecular refractivity (MR), atom counts, and polar surface area (PSA), were computed. This was facilitated by Open Babel, version 2.3.0 (O'Boyle et al., 2011). Evaluation of drug‐likeness candidacy was performed, according to Lipinski (2001), Ghose et al. (1999), Veber et al. (2002), Egan et al. (2000), and Muegge et al. (2001) rules of 5 (RO5) screening. Abbott Bioavailability scores were calculated to predict the likelihood of a compound to possess at least 10% oral bioavailability, considering total charge, topological polar surface area (TPSA), and adherence to Lipinski's filter. Lipophilicity was assessed using iLOGP, XLOGP3, WLOGP, MLOGP, and SILICOS‐IT models, from which a consensus logarithm of n‐octanol/water partition coefficient (log Po/w) value was derived (Daina et al., 2017). Solubility (log S) of the selected ligands was determined using three distinct models: ESOL (Delaney, 2004), Ali (Ali et al., 2012), and SILICOS‐IT (Daina et al., 2017).

### 2.7 Statistical analysis

To determine the relationship between two variables, correlation coefficients (R) were calculated using the CORREL statistical function in MS Excel software. Data are expressed as mean ± SD obtained from three separate observations. The analysis of the data was conducted using SPSS 21.

## 3 RESULTS

### 3.1 Comparison of extraction methods for phenolic and antioxidant content in *S. sclarea* flower extracts

In this study, the antioxidant activity and the total phenolic, flavonoid, flavonol, and tannin contents of extracts obtained from the flower parts of the *S. sclarea* plant were evaluated. The effects of different extraction methods were examined: RE and ME. The results showed that the *S. sclarea* extract obtained by the ME method had higher antioxidant activity (DPPH IC_50_: 260.70 ± 65.41 mg/mL). This value was lower than that of the extract obtained by rotary evaporation (DPPH IC_50_: 345.48 ± 27.91 mg/mL), indicating a stronger antioxidant activity. Additionally, the extract obtained by the ME method had higher values in terms of total phenolic (26.4 ± 7.78 mg GAE/g extract), flavonoid (10.44 ± 0.21 mg QE/g extract), flavonol (9.20 ± 0.84 mg QE/g extract), and tannin contents (8.36 ± 0.39 mg GAE/g extract). In contrast, the extract obtained by RE showed lower values in terms of total phenolic (19.8 ± 3.31 mg GAE/g extract), flavonoid (10.35 ± 0.35 mg QE/g extract), flavonol (5.45 ± 0.01 mg QE/g extract), and tannin contents (7.72 ± 0.10 mg GAE/g extract) (Table 1).

**TABLE 1:** 2,2‐diphenyl‐1‐picrylhydrazyl (DPPH) radical scavenging activities of *S. sclarea* extracts (IC_50_ (μg/mL) ± SD) and total phenolic, flavonoid, flavonol, and tannin contents ± SD* values.

Plant name	DPPH (IC_50_ mg/mL)	Total flavonol compound (mg QE/g extract)	Total flavonoid compound (mg QE/g extract)	Total phenolic compound (mg GAE/g extract)	Total tannin content (mg GAE/g extract)
*S. sclarea* (RE)	345.48 ± 27.91	5.45 ± 0.01	10.35 ± 0.35	19.8 ± 3.31	7.72 ± 0.10
*S. sclarea* (ME)	260.70 ± 65.41	9.20 ± 0.84	10.44 ± 0.21	26.4 ± 7.78	8.36 ± 0.39

* Standard deviation

**FIGURE 2:**
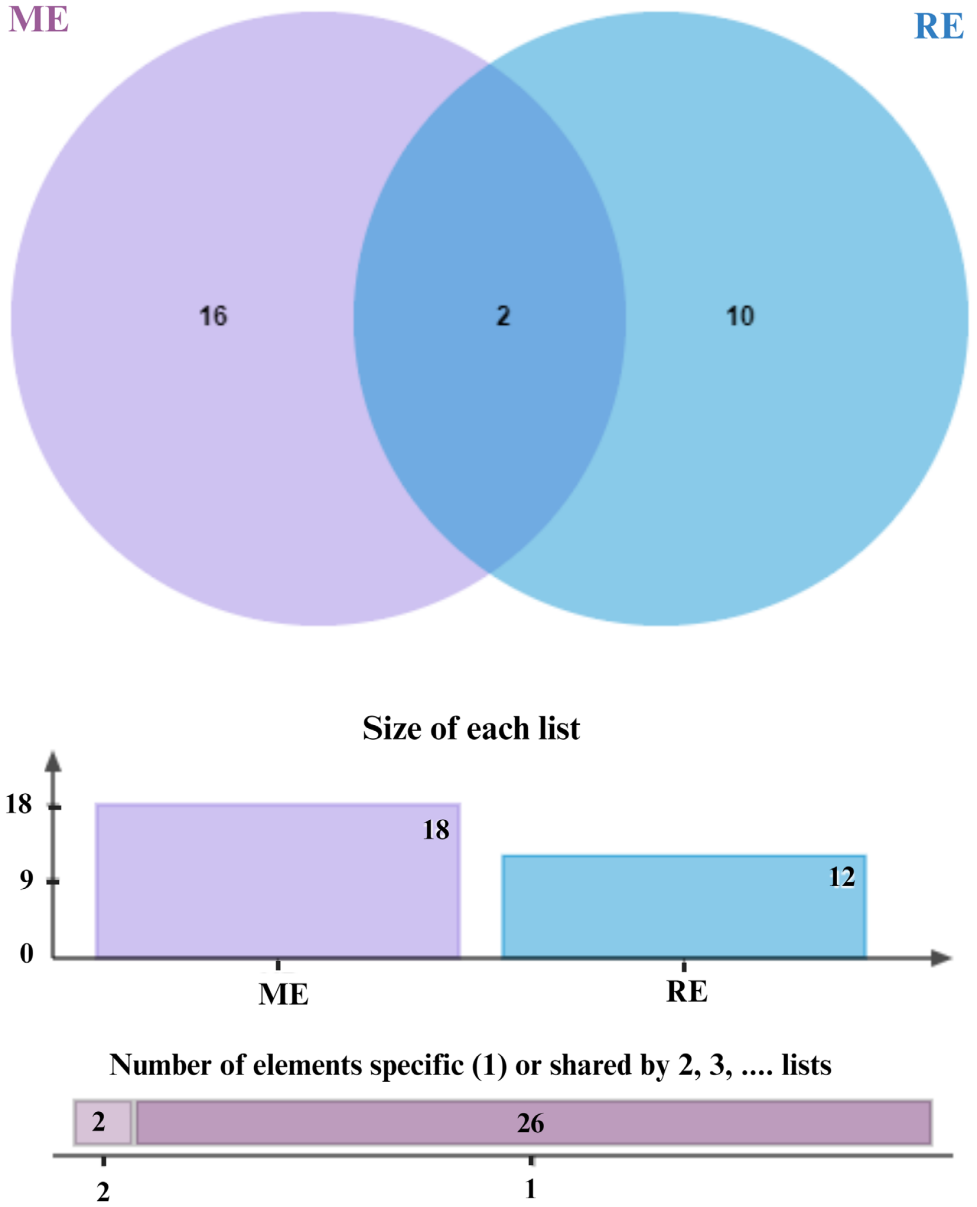
Venn diagram of compounds based on maceration extraction (ME) and rotavapor extraction (RE) GC–MS results.

The flower parts of *S. sclarea* were analyzed using the ME and RE methods with GC–MS technique. Various compounds, such as eucalyptol (2.40%), Terpinen‐4‐ol (2.31%), and cyclononasiloxane, octadecamethyl‐ (2.49%), were identified in the maceration of *S. sclarea* flower parts. Additionally, the extract obtained through the RE method revealed diverse compounds, including N,N‐dimethyl‐4‐nitroso‐3‐(trimethylsilyl)aniline (3.44%), octasiloxane, 1,1,3,3,5,5,7,7,9,9,11,11,13,13,15,15‐hexadecamethyl‐ (2.10%), and hexasiloxane, 1,1,3,3,5,5,7,7,9,9,11,11‐dodecamethyl (2.56%). Furthermore, major components identified in the ME method were tetracosamethyl‐cyclododecasiloxane (34.02%), cyclononasiloxane, octadecamethyl‐ (6.42%), and 1,3,5,7‐tetraethyl‐1,7‐dibutoxytetrasiloxane (5.53%). In the RE, major compounds were tetracosamethyl‐cyclododecasiloxane (27.62%), trimethylsilyl 3‐methyl‐4‐[(trimethylsilyl) oxy] benzoate (8.44%), and cyclononasiloxane, octadecamethyl (3.75%). GC–MS detected the presence of bioactive components in *S. sclarea* extracts. In this study, bioactive metabolites obtained through ME and RE were identified. These bioactive compounds are depicted in Tables 2 and 3. Additionally, the content of the extract obtained through maceration and RE in the study conducted with these two different extraction methods is illustrated in the schematic diagram in Figure 2.

**TABLE 2:** Gas chromatography–mass spectrometry (GC–MS) results of *S. scleara* maceration extraction.

No	RT (min)	Name of the compound	Molecular weight	Area %	Structure
1	6.903	Eucalyptol	154.25	2.40	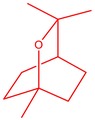
2	7.398	1,2‐Hydrazinedicarboxamide	122.06	0.46	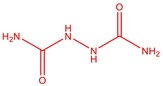
3	15.446	Terpinen‐4‐ol	154.25	2.31	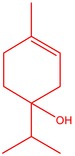
4	49.788	8,11,14‐Docosatrienoic acid, methyl ester	348.60	0.45	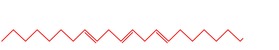
5	50.135	Cyclooctasiloxane, hexadecamethyl‐	593.25	2.49	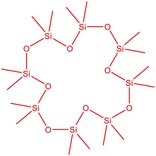
6	51.238	Trimethylsilyl 3‐methyl‐4‐[(trimethylsilyl)oxy] benzoate	296.50	1.56	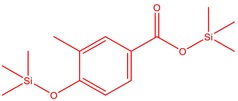
7	51.152	1,3‐Benzenedicarboxylic acid, bis(2‐ethylhexyl) ester	390.55	1.12	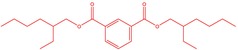
8	51.523	Ethyl 2‐[3‐(3,5‐Dinitrobenzo‐yl) thiol‐ureido] benzoate	418.38	0.52	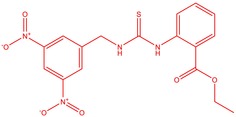
9	51.317	Heptasiloxane, hexadecamethyl‐	533.10	0.51	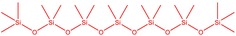
10	51.350	Cyclononasiloxane, octadecamethyl‐	667.40	6.42	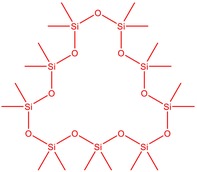
11	52.087	Silicic acid, diethyl bis(trimethylsilyl) ester	296.58	2.58	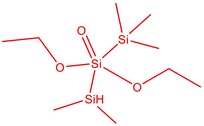
12	52.148	Octasiloxane, 1,1,3,3,5,5,7,7,9,9,11,11,13,13,15,15‐hexadecamethyl‐	577.20	1.29	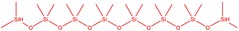
13	52.429	7,7,9,9,11,11‐Hexamethyl‐3,6,8,10,12,15‐hexaoxa‐7,9,11‐trisilaheptadecane	384.69	3.46	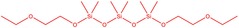
14	52.581	Trimethylsilyl 3‐methyl‐4‐[(trimethylsilyl)oxy] benzoate	296.50	3.75	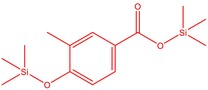
15	53.052	1,3,5,7‐Tetraethyl‐1,7‐dibutoxytetrasiloxane	344.65	5.53	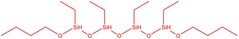
16	53.209	Benzoic acid, 2,3‐bis[(trimethylsilyl)oxy]‐, trimethylsilyl ester	370.66	1.91	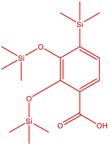
17	53.955	Tetracosamethyl‐cyclododecasiloxane	889.80	34.02	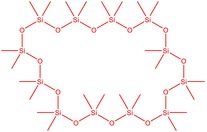
18	54.950	3,4‐Dihydroxymandelic acid, ethyl ester, tri‐TMS	428.70	0.49	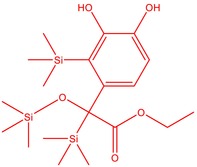

**TABLE 3:** The GC–MS results of *S. scleara* rotavapor extraction (RE).

No	RT (min)	Name of the compound	Molecular weight	Area %	Structure
1	51.160	1,3‐Benzenedicarboxylic acid, bis(2‐ethylhexyl) ester	390.55	0.89	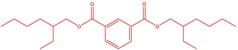
2	51.667	N, N‐Dimethyl‐4‐nitroso‐3‐(trimethylsilyl)aniline	222.36	3.44	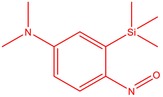
3	52.582	Trimethylsilyl 3‐methyl‐4‐[(trimethylsilyl)oxy] benzoate	296.50	8.44	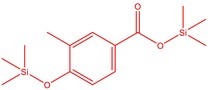
4	52.755	1,2‐Bis(trimethylsilyl)benzene	222.47	1.55	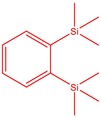
5	52.880	1,2‐Ethanediamine, N‐(2‐aminoethyl)‐	103.16	0.74	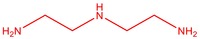
6	53.118	Octasiloxane, 1,1,3,3,5,5,7,7,9,9,11,11,13,13,15,15‐hexadecamethyl‐	390.55	2.10	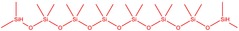
7	53.347	Hexasiloxane, 1,1,3,3,5,5,7,7,9,9,11,11‐dodecamethyl	428.92	2.56	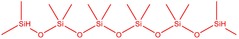
8	53.701	Squalene	422.80	1.50	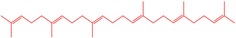
9	54.052	Cyclodecasiloxane, eicosamethyl‐	741.50	1.93	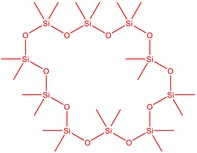
10	54.253	2‐(5‐Methyl‐pyrazol‐1‐yl)‐ethylamine	125.17	1.41	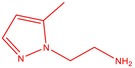
11	55.178	Tetracosamethyl‐cyclododecasiloxane	889.80	27.62	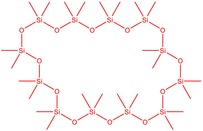
12	55.809	Cyclononasiloxane, octadecamethyl‐	667.40	3.75	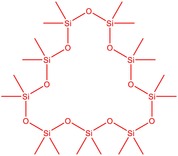

### 3.2 Molecular docking studies

**FIGURE 3:**
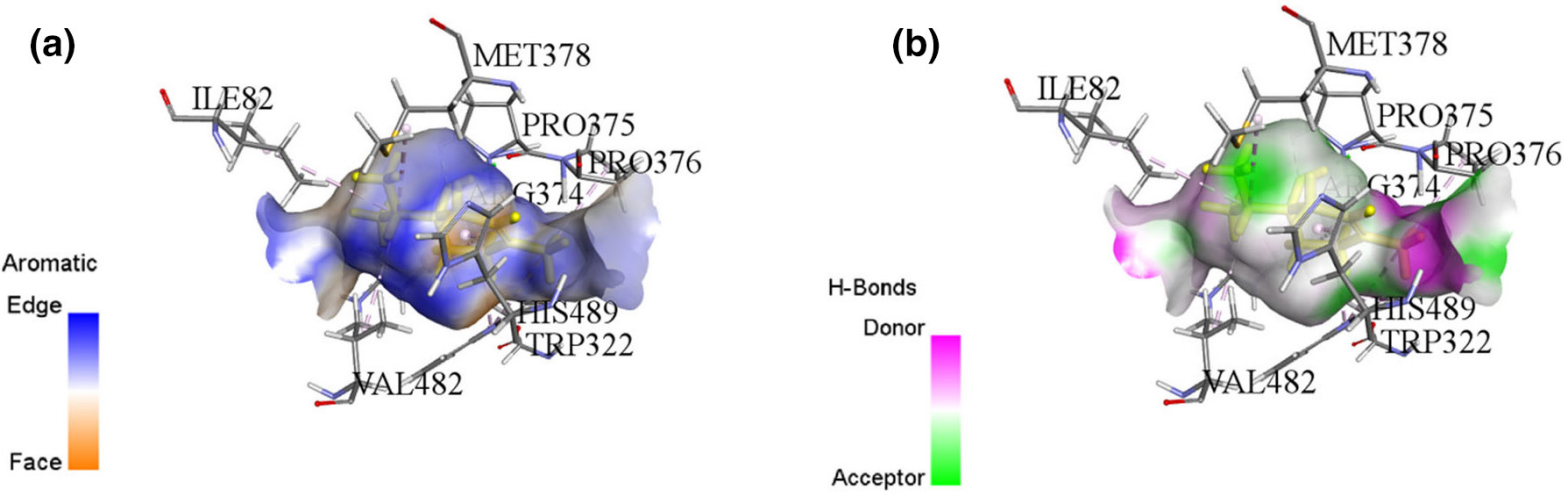
Interaction diagram of Terpinen‐4‐ol (maceration extraction) with 3LD6 proteins in terms of (a) aromatic and (b) H‐bond.

**FIGURE 4:**
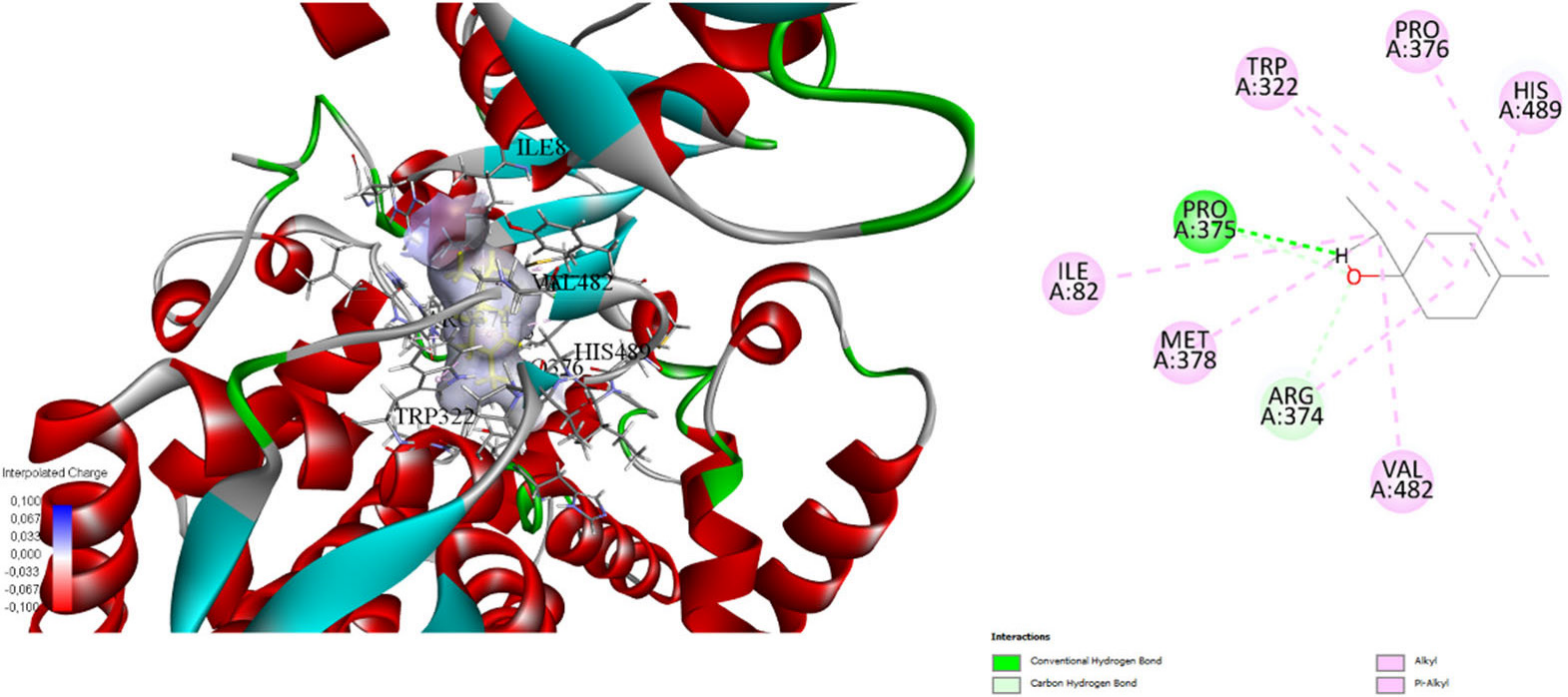
Molecular docking process of Terpinen‐4‐ol with human lanosterol 14α‐demethylase (CYP51) in complex with ketoconazole (3LD6).

**FIGURE 5:**
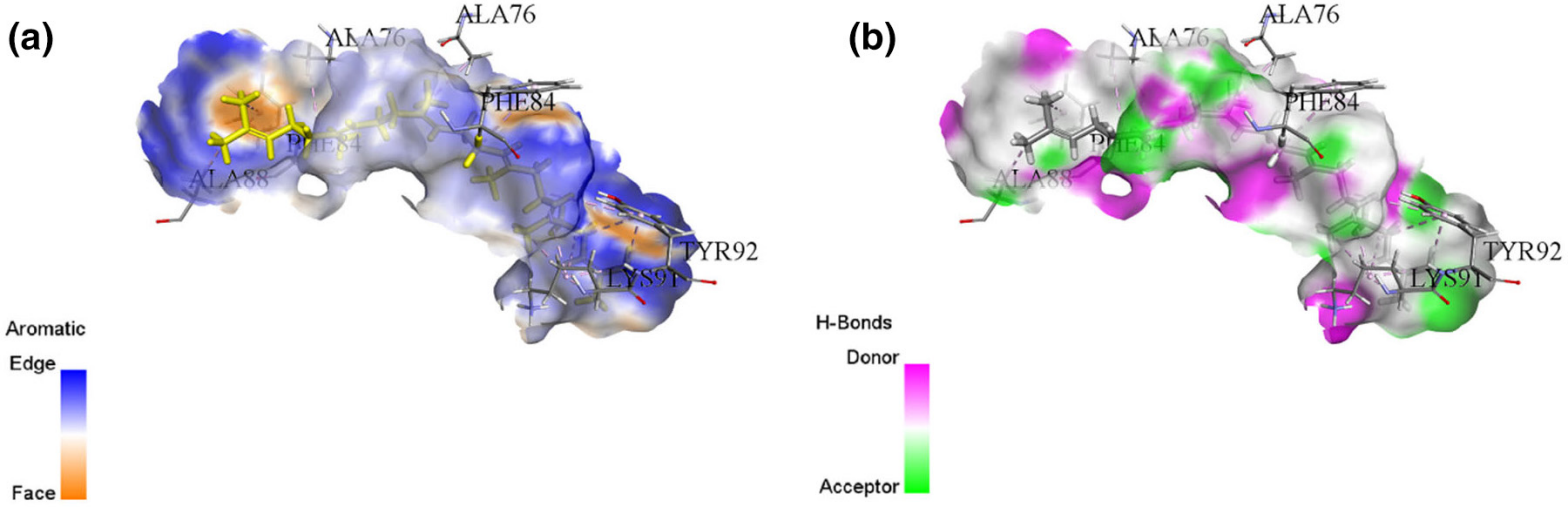
Interaction diagram of Squalene (Rotavapor extraction) with 3LD6 proteins in terms of (a) aromatic and (b) H‐bond.

**FIGURE 6:**
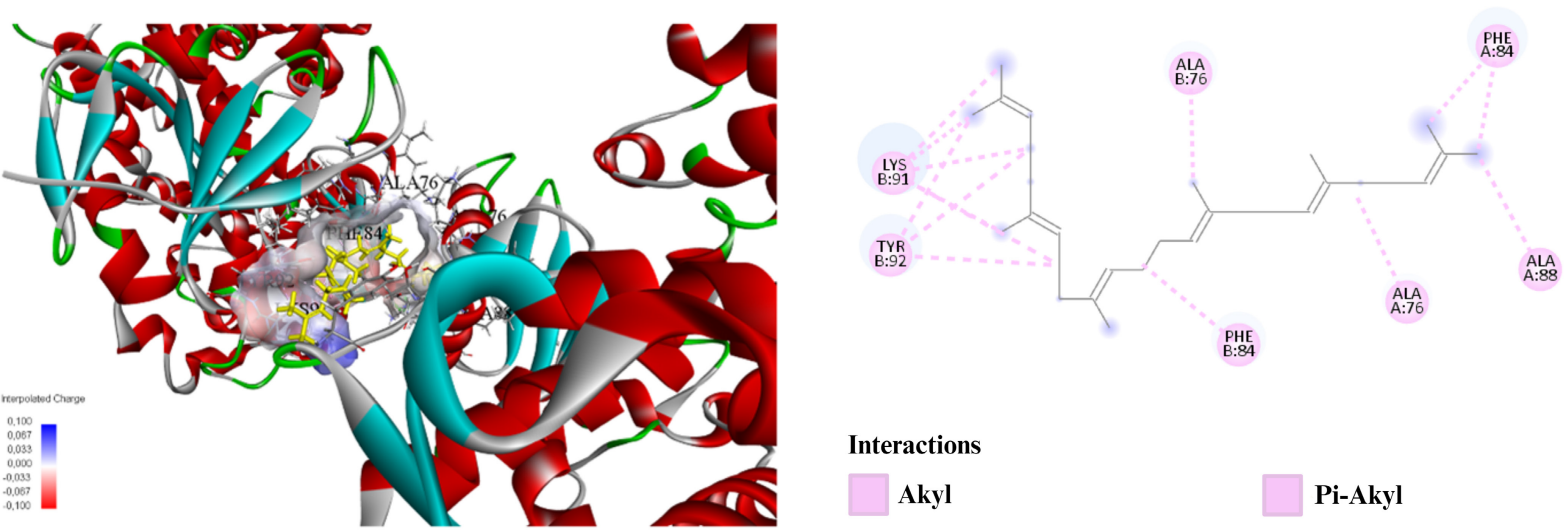
Molecular docking process of Squalene with human lanosterol 14α‐demethylase (CYP51) in complex with ketoconazole (3LD6).

This study examines the binding affinities and interactions of Terpinen‐4‐ol and Squalene compounds with a specific protein target. The two compounds were obtained using different extraction methods, and molecular docking analyses were performed. These analyses include each compound's binding energy, docking score, interacting amino acids, and interaction distances (Table 4). Terpinen‐4‐ol, obtained through the maceration extraction (ME) method, has a binding energy of −6.6 kcal/mol and a docking score of −138.374. Terpinen‐4‐ol exhibits various alkyl and pi–alkyl interactions with amino acids: ALA76 (4.98 Å), ALA88 (4.06 Å), ALA76 (3.54 Å), LYS91 (4.84 Å), LYS91 (4.43 Å), LYS91 (3.46 Å), and LYS91 (4.03 Å) for alkyl interactions, and PHE84 (4.06 Å), PHE84 (4.42 Å), PHE84 (4.26 Å), TYR92 (5.35 Å), and TYR92 (4.12 Å) for pi–alkyl interactions. These results demonstrate that Terpinen‐4‐ol forms strong and diverse interactions with the protein target, indicating a high binding affinity. On the other hand, Squalene, obtained through rotavapor extraction, also has a binding energy of −6.6 kcal/mol, but its docking score is −71.962, which is higher. Squalene exhibits significant hydrogen bonding and alkyl/pi–alkyl interactions. Notable interactions include a conventional hydrogen bond with PRO375:O (1.76 Å) and carbon hydrogen bonds with ARG374:HA:O1 (2.50 Å) and PRO375:HD1:O1 (2.66 Å). Additional alkyl interactions occur with ARG374 (4.79 Å), ILE82 (5.46 Å), MET378 (4.92 Å), VAL482 (4.49 Å), and PRO376 (4.24 Å), and pi–alkyl interactions with TRP322 (5.29 Å), TRP322 (5.20 Å), and HIS489 (5.43 Å). These interactions suggest that Squalene enhances binding stability with the protein target (Figures 3, 4, 5, 6).

**TABLE 4:** Docking scores and report of predicted interactions of docked conformations of compounds against human lanosterol 14α‐demethylase (CYP51) in complex with ketoconazole (3LD6).

Component	Binding energy (kcal/mol)	Docking score	Amino acid	Interacting	Distance
Terpinen‐4‐ol (maceration extraction)	−6.6	−138.374	A: ALA76	Alkyl	4.98
			A: ALA88:C27	Alkyl	4.06
			B: ALA76:C29	Alkyl	3.54
			B: LYS91	Alkyl	4.84
			B: LYS91:C9	Alkyl	4.43
			B: LYS91:C10	Alkyl	3.46
			B: LYS91:C11	Alkyl	4.03
			A: PHE84:C26	Pi–Alkyl	4.06
			A: PHE84:C27	Pi–Alkyl	4.42
			B: PHE84	Pi–Alkyl	4.26
			B: TYR92	Pi–Alkyl	5.35
			B: TYR92:C9	Pi–Alkyl	4.12
Squalene (Rotavapor extraction)	−6.6	−71.962	A: PRO375:O:H18	Conventional hydrogen bond	1.76
			A: ARG374: HA:O1	Carbon hydrogen bond	2.50
			A: PRO375:HD1:O1	Carbon hydrogen bond	2.66
			A: ARG374	Alkyl	4.79
			A: ILE82:C2	Alkyl	5.46
			A: MET378:C2	Alkyl	4.92
			A: VAL482:C2	Alkyl	4.49
			A:PRO376:C10	Alkyl	4.24
			A: TRP322	Pi–Alkyl	5.29
			A: TRP322:C10	Pi–Alkyl	5.20
			A: HIS489	Pi–Alkyl	5.43

In conclusion, Terpinen‐4‐ol demonstrates higher binding affinity with a lower docking score and diverse interactions, whereas Squalene shows stable binding supported by hydrogen bonds and alkyl/pi–alkyl interactions. These findings suggest that Terpinen‐4‐ol could be a more effective inhibitor for this protein target, while Squalene may also be effective due to its specific interactions. Such analyses provide valuable insights into the biological activity of these compounds and lay the groundwork for further experimental studies.

### 3.3 Predicted pharmacokinetics (ADME) parameters of screened compounds

The predicted pharmacokinetic (ADME) parameters for Terpinen‐4‐ol and Squalene provide insights into their absorption, distribution, metabolism, and excretion properties. Terpinen‐4‐ol, obtained through the maceration extraction method, exhibits high gastrointestinal (GI) absorption, indicating an efficient oral uptake. It can permeate the blood–brain barrier (BBB), suggesting potential central nervous system (CNS) activity (Table 5). Terpinen‐4‐ol is not a substrate for P‐glycoprotein (Pgp), which could enhance its intracellular efficacy by preventing expulsion from cells. Importantly, Terpinen‐4‐ol does not inhibit major cytochrome P450 enzymes (CYP1A2, CYP2C19, CYP2C9, CYP2D6, and CYP3A4), indicating a lower risk of drug–drug interactions and hepatic metabolism issues. The compound also has a logarithmic skin permeability (log Kp) value of −5.41 cm/s, reflecting its skin permeability characteristics. In contrast, Squalene, obtained through rotavapor extraction, demonstrates low GI absorption, which may limit its oral bioavailability. It does not permeate the BBB, reducing the likelihood of central nervous system effects. Like Terpinen‐4‐ol, Squalene is not a Pgp substrate, potentially aiding its cellular retention. Like Terpinen‐4‐ol, Squalene does not inhibit the major cytochrome P450 enzymes, suggesting a favorable profile in terms of drug metabolism and interaction potential. Squalene's log Kp value is −0.58 cm/s, indicating different skin permeability properties compared to those of Terpinen‐4‐ol. These pharmacokinetic profiles suggest that Terpinen‐4‐ol may be more suitable for applications requiring high oral absorption and central nervous system penetration, while Squalene's pharmacokinetic characteristics might limit its oral efficacy but could be advantageous for other routes of administration or therapeutic targets. Such analyses provide valuable insights into the biological activity of these compounds and lay the groundwork for further experimental studies.

**TABLE 5:** Predicted pharmacokinetics (ADME) parameters of the screened compounds.

	Terpinen‐4‐ol	Squalene
GI absorption	High	Low
BBB permeant	Yes	No
Pgp substrate	No	No
CYP1A2 inhibitor	No	No
CYP2C19 inhibitor	No	No
CYP2C9 inhibitor	No	No
CYP2D6 inhibitor	No	No
CYP3A4 inhibitor	No	No
Log Kp [cm/s]	−5.41	−0.58

### 3.4 Predicted pharmacokinetics (ADME) parameters of screened compounds

These data encompass various parameters to evaluate the pharmacokinetic properties, drug‐likeness, medicinal chemistry, and lead‐likeness of the investigated compounds, Terpinen‐4‐ol and Squalene. For Terpinen‐4‐ol, it is observed that there are no violations of the Lipinski rule, 2 violations in the Ghose rule, 0 violations in the Veber rule, 0 violations in the Egan rule, and 2 violations in the Muegge rule (Table 6). This indicates that while most pharmacokinetic properties of Terpinen‐4‐ol are acceptable, some aspects require improvement. Additionally, Terpinen‐4‐ol has a bioavailability score of 0.55 and does not exhibit any undesirable properties, such as the pan‐assay interference compounds (PAINS) alerts or Brenk alerts, although 1 violation is observed in terms of lead‐likeness. The synthetic accessibility score is determined as 3.05, total polar surface area (TPSA) as 20.23 A^2, and the logP value (cLogP) as 2.43. In the case of Squalene, there is a violation of the Lipinski rule, 3 violations in the Ghose rule, 1 violation in the Veber rule, 1 violation in the Egan rule, and 2 violations in the Muegge rule. This suggests that the pharmacokinetic properties of Squalene are less favorable and may have certain properties that could limit its potential as a drug. The bioavailability score is 0.55, and no PAINS alerts or Brenk alerts are detected, although 3 violations are observed in terms of lead‐likeness. The synthetic accessibility score is determined as 4.73, the total polar surface area (TPSA) as 0 A^2, and the logP value (cLogP) as 9.38. These data indicate that Terpinen‐4‐ol exhibits more favorable pharmacokinetic properties, although improvement may be needed in terms of synthetic accessibility. On the other hand, Squalene shows less favorable pharmacokinetic properties and more violations in terms of lead‐likeness. Both compounds may have potential usefulness in certain pharmacological aspects, but factors such as synthetic accessibility and lead‐likeness need to be considered.

**TABLE 6:** Predicted drug‐likeness, medicinal chemistry, and lead‐likeness pharmacokinetics parameters of the screened compounds.

	Terpinen‐4‐ol	Squalene
Lipinski	0	1
Ghose	0	3
Veber	0	1
Egan	0	1
Muegge	2	2
Bioavailability score	0.55	0.55
PAINS	0	0
Brenk	0	1
Lead‐likeness	1	3
Synthetic accessibility	3.05	4.73
TPSA	20.23 A^2^	0
LogP(cLogP)	2.43	9.38

## 4 DISCUSSION

The phenolic compounds are an important group of compounds found in nature and possess strong antioxidant properties (Shahidi et al., 1992). Their occurrence in plants holds significance as polyphenolic compounds, characterized by hydroxyl groups, demonstrate a robust capacity to effectively scavenge free radicals (Villano et al., 2007). Flavonoids, a specific subgroup within polyphenolic compounds, showcase a diverse range of biological effects. These effects include antioxidant properties, antiulcer activity, anticancer potential, anti‐inflammatory effects, and hepatoprotective benefits (Umamaheswari and Chatterjee, 2008). Flavonoids, equipped with phenolic hydroxyl groups, demonstrate a high efficacy in scavenging reactive oxygen species, showcasing their robust antioxidant potential (Cao et al., 1997).

The obtained results indicate that the plant extracts obtained by the ME method in our study exhibit high antioxidant activity and contain significant levels of total phenolic, tannin, flavonol, and flavonoid contents. Numerous research works have shown that *S. sclarea* extract possesses high antioxidant activity and is rich in total phenolic, flavonol, flavonoid, and tannin contents. According to the analysis performed by Sharopov et al. (2018), the IC_50_ value for the DPPH radical scavenging effect of RE extracts from *S. sclarea* leaves, stems, and flowers was found to be 21.6 ± 1.3 μg/mL, while the root IC_50_ value was 65.9 ± 5.3 μg/mL. The determined total phenolic content in *S. sclarea* leaves, stems, and flowers was 1771.9 mg GAE/g, whereas the roots exhibited a total phenolic content of 658.3 mg GAE/g. Additionally, the total flavonoid content in *S. sclarea* leaves, stems, and flowers was found to be 95.4 QE/g, whereas the roots showed a total flavonoid content of 13.0 QE/g (Sharopov et al., 2018). In the study conducted by Firuzi et al. (2013), the IC_50_ value for the DPPH radical scavenging effect of RE extracts from *S. sclarea* leaves, stems, and flowers was found to be 190.74 ± 15.7 μg/mL. The total phenolic content in *S. sclarea* leaves, stems, and flowers was determined to be 14.83 ± 0.80 GAE/kg (Firuzi et al., 2013). According to the study by Loizzo et al. (2014), the IC_50_ value for the DPPH radical scavenging effect of the RE extract from the aerial parts of *S. sclarea* was 4.8 ± 0.1 μg/mL, and the total phenolic content in the aerial parts was 30.4 ± 0,3 mg GAE/g (Loizzo et al., 2014). In the study by Zengin, Atasagun, et al. (2019), the total phenolic content of *Salvia modesta* methanol extract was reported as 60.50 ± 0.86 mg GAE/g, the dichloromethane extract as 24.18 ± 1.23 mg GAE/g, and the water decoction as 78.63 ± 1.51 mg GAE/g. Additionally, the total flavonoid content of the methanol extract was found to be 27.64 ± 0.50 mg RE/g, the water decoction 26.91 ± 0.33 mg RE/g, and the dichloromethane extract 14.73 ± 0.97 mg RE/g (Zengin, Atasagun, et al., 2019).

In the study by Kamatou et al. (2010), the IC50 values for the DPPH radical scavenging effect of *Salvia* spp. RE extracts were reported as follows: *S. caerulea* 33.4 ± 3.7 mg/mL, *S. lutea* 47.6 ± 2.61 mg/mL, *S. albicaulis* 19.9 ± 1.01 mg/mL, *S. aurita* 16.6 ± 0.2 mg/mL, *S. chamelaeagnea* 12.8 ± 1.04 mg/mL, *S. disermas* 55.1 ± 4.01 mg/mL, *S. garipensis* 74.2 ± 2.85 mg/mL, *S. lanceolata* 68.1 ± 3.69 mg/mL, *S. muirii* 11.1 ± 0.52 mg/mL, *S. namaensis* 0.6 ± 0.75 mg/mL, *S. repens* 15.5 ± 1.75 mg/mL, *S. runcinata* 9.3 ± 0.61 mg/mL, *S. schlechteri* 1.61 ± 0.03 mg/mL, and *S. stenophylla* 14.9 ± 0.93 mg/mL (Kamatou et al., 2010). In the study by Aghaei Jeshvaghani et al. (2015), the IC_50_ values for DPPH radical scavenging effect of *Salvia* spp. ME extracts are reported as follows: 473.2 mg/mL for *S. nemorosa*, 198.0 mg/mL for *S. virgata*, 290.0 mg/mL for *S. sclarea*, 233.0 mg/mL for *S. officinalis*, 598.0 mg/mL for *S. reuterana*, 883.0 mg/mL for *S. cereal*, and 1810.0 mg/mL for *S. persica*. The total phenolic content of the ME ranges from 33.83 to 114 mg tannic acid/1 g of dry plant. The highest total phenolic content is obtained in *S. nemorosa* (114 mg TAE/1 g DW), followed by *S. sclarea* (103.3 mg TAE/1 g DW) and *S. officinalis* (86.4 mg TAE/1 g DW), while the lowest content is obtained in the methanolic extract of *S. virgata* (33.83 mg TAE/1 g) (Aghaei Jeshvaghani et al., 2015). In the study by Zengin, Mahomoodally, et al. (2019), the ultrasonic‐assisted extraction of *Salvia viridis* ethanolic root extracts was reported to have a total phenolic content of 111.41 mg GE/g extract and a total flavonoid content of 23.46 mg RE/g extract. Additionally, the *S. viridis* ethanolic root extract exhibited the highest radical scavenging activity, with a DPPH value of 240.00 mg TE/g (Zengin, Mahomoodally, et al., 2019). In the study by Zengin, Llorent‐Martínez, et al. (2018), the methanol extract of *S. sclarea* was found to have a total phenolic content of 81.78 mg GAE/g extract and a total flavonoid content of 40.59 mg RE/g extract. Molecular docking studies revealed that quercetin binds to tyrosinase via two hydrogen bonds and one π–π bond (Zengin, Senkardes, et al., 2018).

In the conducted studies, RE method was preferred more for obtaining samples, whereas the utilization of ME method was found to be more limited.

The compound Eucalyptol, present in the hydroalcoholic extract (HAE) of the flower parts of *S. sclarea*, exhibits antioxidant and anti‐inflammatory properties (Kennedy‐Feitosa et al., 2019), Terpinen‐4‐ol reveals antibacterial activity (Kamiya et al., 2023), cyclononasiloxane, octadecamethyl‐ possesses antifungal activity (Syed et al., 2022). The compound tetracosamethyl‐cyclododecasiloxane possesses anti‐inflammatory, hypocholesterolemic, anticancer, and antibiofilm properties (Gebreyohannes & Sbhatu, 2023). The compound N,N‐dimethyl‐4‐nitroso‐3‐(trimethylsilyl)aniline, present in the ME of flower parts of *S. sclarea*, exhibits anticancer properties (Ryan et al., 2007), octasiloxane, 1,1,3,3,5,5,7,7,9,9,11,11,13,13,15,15‐hexadecamethyl‐ reveals insecticidal activity (Farag et al., 2021). Hexasiloxane, 1,1,3,3,5,5,7,7,9,9,11,11‐dodecamethyl, possesses significant biological activities, such as antimicrobial, antioxidant, and anti‐inflammatory properties (Bopi et al., 2019). Squalene reveals antioxidant (Zhang, Liu, et al., 2023) and antibacterial (Fang et al., 2019) properties.

Our study demonstrates that extracts obtained using the HAE method exhibit high antioxidant activity and contain significant levels of total phenolics, tannins, flavanols, and flavonoids. Additionally, it has been shown that extracts obtained through the HAE method contain various compounds and result in the acquisition of a greater variety of components. This study aims to elucidate the differences in secondary metabolites resulting from different extraction methods and the potential biological interactions arising from these differences through molecular docking studies.

## 5 CONCLUSION

When compared to rovapotor extraction, maceration extraction of *S. sclarea* has been determined to exhibit higher phenolic content and higher antioxidant activities. The results obtained from different methods shed light on the potential pharmacological uses of this plant. The antioxidant properties of *S. sclarea* hold promise for therapeutic potentials. Additionally, Terpinen‐4‐ol identified in maceration extraction (ME) and Squalene compounds identified in rotavapor extraction have shown a high affinity for the Sterol 14α‐demethylase cytochrome P450 (CYP51) protein. Various drugs targeting the CYP51 gene are available to combat *Candid*a infections. *S. sclarea* has demonstrated significant antifungal activity in conducted studies. The compounds potentially suitable for the treatment of human trypanosomiasis, by inhibiting the CYP51 gene, underscore the importance of developing new therapeutic options. Consequently, these compounds hold high direct therapeutic potential.

## AUTHOR CONTRIBUTIONS

**Emine Incilay Torunoglu:** Investigation (equal); methodology (equal); supervision (equal); validation (equal); writing – original draft (equal); writing – review and editing (equal). **Erdi Can Aytar:** Investigation (equal); methodology (equal); validation (equal); visualization (equal); writing – original draft (equal). **Alper Durmaz:** Data curation (equal).

## CONFLICT OF INTEREST STATEMENT

The authors declare no conflicts of interest.

## CONSENT TO PARTICIPATE

All authors agreed to participate in this study.

## CONSENT TO PUBLISH

All authors agreed to publish the data in this study.

## Data Availability

All the datasets generated or analyzed during this study are included in this published article.
